# Development of a Colorimetric Sensor for Autonomous, Networked, Real-Time Application

**DOI:** 10.3390/s20205857

**Published:** 2020-10-16

**Authors:** Brandy J. Johnson, Anthony P. Malanoski, Jeffrey S. Erickson

**Affiliations:** Center for Bio/Molecular Science & Engineering, US Naval Research Laboratory, Washington, DC 20375, USA; anthony.malanoski@nrl.navy.mil (A.P.M.); jeffrey.erickson@nrl.navy.mil (J.S.E.)

**Keywords:** reflectance, portable, autonomous, sensor, porphyrin, color value, chemical detection, environmental monitoring

## Abstract

This review describes an ongoing effort intended to develop wireless sensor networks for real-time monitoring of airborne targets across a broad area. The goal is to apply the spectrophotometric characteristics of porphyrins and metalloporphyrins in a colorimetric array for detection and discrimination of changes in the chemical composition of environmental air samples. The work includes hardware, software, and firmware design as well as development of algorithms for identification of event occurrence and discrimination of targets. Here, we describe the prototype devices and algorithms related to this effort as well as work directed at selection of indicator arrays for use with the system. Finally, we review the field trials completed with the prototype devices and discuss the outlook for further development.

## 1. Introduction

This review addresses the development of a sensor system intended to offer utility in a wireless sensor network for real-time monitoring of airborne chemical targets. These devices are intended to offer a portable and low-power solution for real-time detection of small molecule chemical threats in indoor and outdoor environments. Past efforts to detect such threats have applied a variety of techniques, including Raman and IR spectroscopy [1,2,3,4,5], gas or liquid chromatography coupled with mass spectroscopy [6,7,8,9], classic wet chemical assays [10,11], and others. For many of these methods, samples are collected at the point of interest and sent to central laboratories for processing. Such strategies can require hours to weeks before results are available, and they are not suitable for real-time protection. Other approaches seek to avoid these limitations through adaptation of devices for field use. The document Strategies to Protect the Health of Deployed U.S. Forces: Detecting, Characterizing, and Documenting Exposures (summarized in their Appendix D) provides a comprehensive list of technologies applied to chemical threat detection and references for detailed information on these applications [12]. Within the US Department of Defense, M8 Chemical Detection Paper and the Joint Chemical Agent Detector (JCAD) offer the most widely used approaches to portable point detection. M8 paper provides detection in a colorimetric format with visual discrimination. The JACD is a handheld, ion mobility spectrometry (IMS) based detector that has been available for more than 15 years [13]. Each of these devices is intended for hand-held use by a technician at the site of potential contamination.

More recently, the distributed microsensor paradigm has emerged as a potential solution to the detection of chemical and biological compounds. Significant research and modeling of plume dispersion, particularly in urban environments, points to the weakness of single detector deployment and the need for a more distributed network [14,15,16,17,18,19,20,21,22,23]. The idea behind this approach is to utilize multiple low-cost sensors that are small, low power, and autonomous [24,25]. The devices could be used with unmanned vehicles for deployment and/or delivery [26,27,28,29,30] or may be scattered around an area of interest for long-duration monitoring. The concept of use involves a population of these sensors rather than a single device. While no individual device would match the sensitivity of traditional laboratory equipment, there are advantages to be found. First, at least one of the devices would be expected to be nearer the point of origin than a centralized detector or detector bearing personnel, resulting in a proportionally larger concentration, and reducing strict sensitivity requirements. Second, a geographical distribution of the devices could also offer spatial and temporal information on the movement of a chemical plume. Finally, detection may occur sooner as threats do not have to diffuse to a centralized detector but will instead encounter devices at the perimeter and throughout the area. Various detection methods have been tested in sensor networks [31,32,33,34,35]. There are several topics that remain active areas of focus, such as controlling power use [31,36,37,38] and determining the best way to deploy the sensors [39,40,41,42].

This review addresses development of a sensor system intended for distributed autonomous applications, the Array-Based Environmental Air Monitor (ABEAM). The ABEAM is a colorimetric array utilizing porphyrin and metalloporphyrin indicators with commercially available color sensing chips. The work includes selection of indicator materials, hardware, firmware, and software design, and development of algorithms for determination of responses. Over several generations of devices, demonstrations have included laboratory-based screening of indicator responses, independent laboratory evaluations, and outdoor deployments for periods of up to months. Here, we will review the key components of the devices as well as specific results for their evaluation.

## 2. Indicators

**Figure 1:**
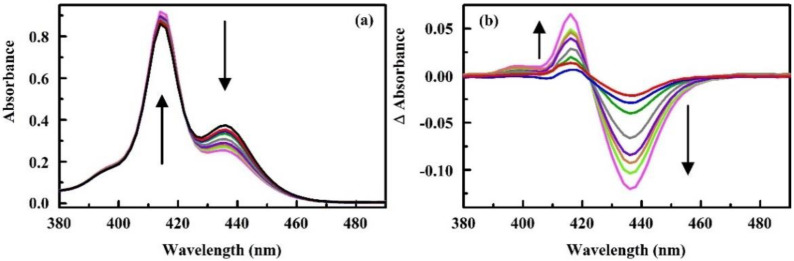
(**a**) Absorbance spectra of C4TPP in the absence and presence of ethanol. (**b**) Difference absorbance spectra (right) are calculated as porphyrin + target minus porphyrin only. C4TPP is 9.86 mM in deionized water (black). Ethanol is 57 (red), 113 (blue), 169 (green), 225 (gray), 280 (purple), 335 (orange), 390 (lime), and 444 mM (pink). Arrows indicate increasing target concentration [78].

The prototype sensors of this review use paper-supported, porphyrin, and metalloporphyrin indicators for detection of chemical targets. Porphyrins offer a large macrocycle, strongly absorbing visible light with the highest extinction coefficients in the blue region of the spectrum. Strong changes in the spectrophotometric characteristics of this class of compounds resulting from changes in the chemical composition of their immediate environment make them well suited to the application described here [43]. These changes are commonly reported as changes in absorbance or fluorescence (as in Figure 1) but can also be interrogated by the reflectance approach utilized by the prototype devices described here.

A wide range of both natural and synthetic porphyrins are available with different porphyrin and metalloporphyrin variants providing different responses to a given target. For example, porphyrins and metalloporphyrins have been widely described for detection of volatile targets based on changes in their spectrophotometric characteristics upon target interaction [44,45,46,47]. These interactions, however, tend to be non-specific with broad ranges of compounds leading to changes in the characteristics of a single indicator. As a result, the use of a single indicator for a specific target is not typical. Utilization as an array with analysis based on a fingerprint response across a number of indicators has been described [44,48,49,50,51,52,53,54,55,56,57].

Porphyrin and metalloporphyrin indicators can also be applied in combination with antimicrobial peptides for a similar approach to detection of biological compounds [58,59,60]. This type of modification of the peripheral porphyrin structure is similar to that used for chemical detection. The difference is in the incorporation of a modification specifically designed to change upon target interaction rather than being designed to closely associate a target with the porphyrin structure. Again, these interactions tend to be non-specific requiring use of an array-based response for discrimination of biological targets.

### 2.1. Porphyrin Based Chemical Detection

The approach taken here for chemical detection is similar to that described by Suslick et al. [44,47,61,62] and others [44,62,63,64,65,66,67] in that reflectance-based color changes are used; however, the indicators are selected for a real-time detection application and tend to yield reversible changes. This is a consideration that also leads to collection and use of a different type of data. Other approaches focus on image processing and automation of this task [44,52,61,68,69,70], including development of algorithms for use with smartphones [71,72,73]. Those types of approaches sample at a time point following a specific exposure duration using detection elements that undergo irreversible changes. The real-time effort described here evaluates a continuous data stream and uses RGB (red, green, blue) color values rather than photographs of the indicator [74].

The porphyrin indicators are supported on WypAll X60 paper using a dip and dry deposition approach optimized based on evaluation of sensor responses for materials with varied porphyrin concentrations [75]. The deposition procedure was designed to produce sufficient porphyrin loading and homogeneous coverage of the support material without overloading the support, a condition that reduces indicator responsiveness [74]. The specific indictors selected were identified using several types of experiments depending on the prototype version in use [74,75,76]. The most commonly used screening experiment used continuous data collection over periods of 1 to 14 days with multiple exposure and purge periods. A glove chamber (65 L; Techni-Dome, Bel-Art, Wayne, NJ, USA) was used to house the devices and provide a controlled environment. The volume was purged with humidified air for baseline conditions. Exposures were accomplished by adding a volume of target to the humidified air stream to produce alcohol concentrations of 0.05, 0.16, 0.32, 0.53, 1.06, and 1.58 ppm.

In addition to evaluation with the prototype devices, a series of porphyrins were evaluated in solution [77,78,79]. The work proposes the use of affinity coefficients in identification of indicators for use in paper supported arrays. Solution binding experiments can be completed rapidly, and large datasets are available in the literature. If the approach provides a valid method for selection of indicators, it would significantly speed the process. These experiments evaluated metalloporphyrin variants of meso-tetra(4-aminophenyl) porphyrin (N4TPP) [77], meso-tetra(4-carboxyphenyl) porphyrin (C4TPP) [78], and meso-tetra(4-sulfonatophenyl) porphyrin (S4TPP) [79] using ethanol, methanol, and isopropanol as the targets (Figure 1). Affinity coefficients were determined based on the intensity changes in the absorbance spectra. The various porphyrins and metalloporphyrins showed varying affinity (over six orders of magnitude) and changes in extinction coefficient upon interaction with the targets. Comparison of these results to those obtained using paper supported materials is ongoing.

### 2.2. Antimicrobial Peptide Based Biological Detection

Antimicrobial peptides (AMPs) are a group of biomolecules that recognize and kill biological targets by binding to and disrupting cell membranes. These compounds are stable under environmental extremes and offer high affinity with overlapping binding interactions. Arrays of these compounds have been applied previously to detection and classification of bacterial and viral targets [80,81,82,83,84]. Optical approaches typically use a surface immobilized antimicrobial peptide in combination with an additional reagent, a tracer of some type. Porphyrin-peptide conjugates have been used previously for targeting of photoactive reagents [85,86,87,88,89]. For the application described here, porphyrin-modified antimicrobial peptides also provide target recognition, but a change in the spectrophotometric signature of the porphyrin upon interaction with that target is used for indication. No additional reagents are necessary. While systems requiring additional reagents are poorly suited to long-term monitoring applications, the porphyrin-modified antimicrobial peptide beacons facilitate the long-term application desired.

**Figure 2:**
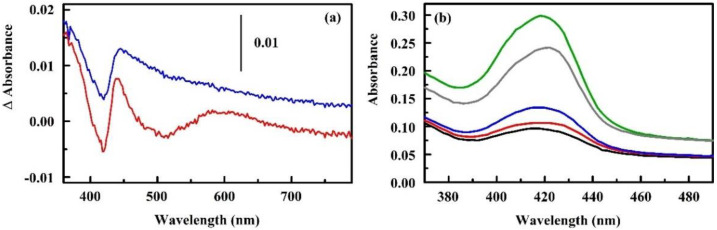
(**a**) Interaction of 5-mono(4-carboxyphenyl)-10, 15, 20-triphenyl porphine (C1TPP) conjugated to the indolicidin antimicrobial peptide with bacterial cells. Absorbance difference spectra are calculated based on the point-by-point subtraction of the spectrum of the construct from that of the construct in the presence of target [58,60]. (**b**) Absorbance characteristics for the C1 end C1S3TPP modified indolicidin (16 mM): *N*-(α-maleimidoacetoxy) succinimide ester (AMAS, black), (*N*-γ-maleimidobutyryl-oxysuccinimide ester) (GMBS, red), *N*-(ε-maleimidocaproyloxy)succinimide ester (EMCS, green), *m*-maleimidobenzoyl-*N*-hydroxysuccinimide ester (MBS, blue), succinimidyl 4-(*N*-maleimidomethyl)cyclohexane-1-carboxylate (SMCC, gray).

An initial study provided proof of concept data for the use of porphyrin-modified antimicrobial peptides for indication of bacterial presence [58,60]. Sensing of the bacteria is a result of changes in the local environment of the covalently attached porphyrin resulting from conformational changes in the antimicrobial peptide. Peptides with little to no change in conformation upon target interaction did not provide changes in absorbance or fluorescence upon exposure to the targets, *Escherichia coli* and *Bacillus cereus* (Figure 2a) [58,60]. Different spectrophotometric changes were observed for constructs based on antimicrobial peptides that do change upon target binding. Addition of coordinated metal to the constructs altered the spectrophotometric characteristics and the noted changes. An initial evaluation of the constructs in a paper-supported format was reported [90], but detailed evaluation and analysis of these materials is still underway.

A series of crosslinker variations were considered for preparation of an indolicidin-based construct using meso-tri(4-sulfonatophenyl)mono(4-carboxyphenyl) porphyrin (C1S3TPP, Figure 2b) [59]. The goal in generation of the constructs was to provide an array in which the peptide components provide target recognition, with the porphyrin providing the detected signal. For the approach to function properly, the spectrophotometric characteristics of the porphyrin should be impacted by the change in the structure of the peptide upon target binding. A direct interaction of the porphyrin with the bacterial target would likely result in nonspecific changes to the spectrophotometric characteristics. The crosslinker series was intended to provide control over the point of modification and the ratio of porphyrin to peptide. The study found that the nonspecific 1-Ethyl-3-(3-dimethylaminopropyl) carbodiimide hydrochloride (EDC)/*N*-hydroxysuccinimidyl ester (NHS)-based chemistry provided constructs with the largest change in spectrophotometric characteristics upon target interaction [59]. Other crosslinkers produced constructs that failed to show spectrophotometric changes or showed only small to moderate changes in characteristics. Work focused on the use of these indicators with the prototype devices is ongoing.

## 3. Devices

A number of reports describing the development of array-based sensing approaches are available [44,61,76,91], including both electrochemical and optical approaches [61,91,92,93,94]. Optical methods can be based on image capture, typically requiring significant post processing, or may be based on simple color intensity measurements [44,52,61,68,69,70]. Commercial portable sensors have been described based on analysis of reflected color or intensity. These devices may use analysis of a spectrum or may employ photodiodes with optical color filters. Breakout boards (i.e., Parallax Color-PAL; Seeed Studio Grove I2C Color Sensor; Hamamatsu C9331) to facilitate these types of applications are available. These types of devices have been used in a limited number of applications previously [95,96].

The earliest work in development of the device described here evaluated the TCS3200-DB color sensing breakout board (Parallax, Inc., Rocklin, CA, USA). This device provides a color light-to-frequency integrated circuit with white LED illumination, and an adjustable lens. The board was used in a reflectance-type measurement with 2.54 cm standoffs used to mount the sensor above an indicator supporting platform. Firmware was developed on an Arduino Uno using a firmware sketch for collection of frequency data and a converter providing RGB numbers and saving to a text file. Briefly, the LEDs were turned on and data were collected in time increments of 1 s for white, red, green, and blue signals.

**Figure 3:**
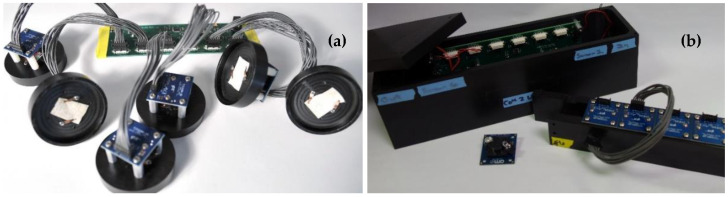
(**a**) Photograph of the Multiplex including custom printed circuit board (PCB) with six TCS3200-DB RGB color sensors and six custom holders attached. Photo: US Naval Research Laboratory/Jamie Hartman. (**b**) Photograph of the ABEAM-6 including custom printed circuit board (PCB) with housing and custom wind tunnel support for color sensors and indicators. Additional detail is provided in the Supplementary Information, Figures S1 and S2.

In order to create a multi-indicator device (Multiplex), a customized multiplex platform was developed [74,97]. This variant utilized six of the TCS3200-DB breakout boards. A custom-printed circuit board (PCB) utilizing an ATMega microcontroller (ATMega 328P, Microchip Technology, San Jose, CA, USA) was designed. This board regulated the timing of events, counted pulses, and reported the results to a computer. Communications between the sensor device and the controlling computer were via USB; power was supplied through a DC barrel jack (Figure 3a). A software-based graphical user interface (GUI) was developed using LabWindows (National Instruments, Austin, TX, USA). Each color sensing board sequentially measured four colors: Red, green, blue, and white. As an example, the microcontroller enabled sensor #1, this turned on the LEDs and initiated communication of data. The timer channel counted the number of pulses received from the white sensor channel and stored the result. The process was repeated three additional times for the red, green, and blue channels, respectively (0.8 s total). Sensor #1 was then disabled, and sensor #2 was enabled to follow the same process. This continued through all six sensors (4.8 s total). Finally, data were sent to the controlling computer. The total time for a single read of the device at 100 ms integration (per channel) was fixed by this process at 5 s. For this device, custom Delrin holders for each of the breakout boards provided the 2.54 cm standoff and supported the indicator material. They were designed to sit as the lid on a 60 mm petri dish to support the indicator material over a liquid sample for vapor testing.

The next device variant (ABEAM-6) added an enclosure and autonomous capabilities to the sensor package allowing for unattended operation and use in outdoor environments (Figure 3b). The housing was machined from Delrin plastic for chemical resistance; black was selected to minimize stray reflections. Holes in the housing for USB connection, power, and fans are located on the bottom to allow for use in outdoor environments. This device included an upgraded microcontroller (XMEGA 64A3U-AU, Microchip Technology, San Jose, CA, USA) and flash memory suitable for deployment durations of greater than 14 days; the original device accommodated only 7 days of data. The device was powered using a 7.5 V power supply (or set of batteries). A PC equipped with custom software (LabWindows, National Instruments, Austin, TX, USA) was used to control the beginning and end of data collection and to download data from the device; connection was not required during data collection. Data were acquired in real time and stored on flash memory.

## 4. Algorithms

In addition to hardware, software, and firmware, algorithms for identifying event occurrence and interpreting indicator responses are necessary for a complete sensor package. As described above, most prior work has focused on image analysis with the sensor device generating images that are processed with a computer using custom software for analysis. For the prototypes described here, the intention was to utilize real-time RGB color values with an algorithm that required minimal processing power for event identification. The aim was to minimize cost of devices as well as reduce the required communication events from the distributed devices to central reporting node. This required significantly different approaches to data handling and determination of event occurrence. Here, we focus on event detection; the classification algorithm is still under development and is likely to reside on the central node and not on the individual devices. The algorithms developed for use with the prototype devices began with exclusion of anomalous data points [75,76,97,98]. Individual RGB values were compared to those reported for the previous time point. The prior value was substituted for the current values, if the absolute value of the difference between the two time points was greater than 35% of the signal. The white channel data were ignored by all algorithms.

### 4.1. Standard Deviation Algorithm

**Figure 4:**
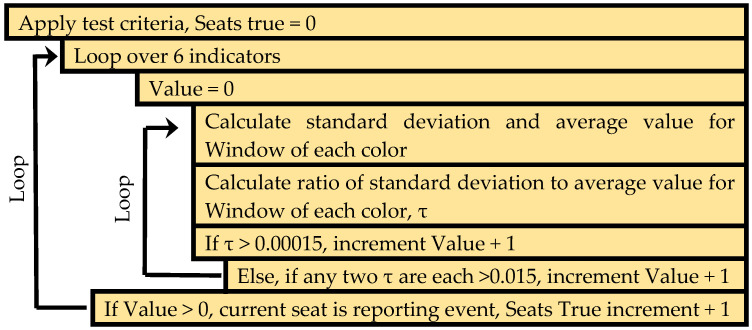
Pseudocode describing the standard-deviation-based algorithm [76].

Initial work utilized a standard deviation based algorithm [76]. For this approach, detection criteria were based on examination of the red, green, and blue color channels for each of the six indicators. For a given time point, the standard deviation for each color channel (RGB) of each color sensor (indicator) was computed using the 12 most recently collected data points. The standard deviations were then divided by the average intensity for the 12 points. If the result of this calculation for the three color channels was greater than 0.00015, the data were determined to represent a potential detection event; similarly, if any two of the values were greater than 0.015, a possible event was identified. For the complete device (all six indicators), a detection event was indicated if the minimum number of indicators reported an event simultaneously; this requirement may be for one to six seats as defined by the application. Once an event has been identified, a cool down window of 30 min was initiated. All detection events within that window were included as part of the initial event, and the window was extended to 30 min past the last set of data meeting detection criteria (Figure 4).

The standard-deviation-based algorithm provided rapid event detection (1 min or less) with minimal instrument warm up time (1 min). When used with the original prototype (Figure 3a), ROC analysis indicated a sensitivity (or true positive rate) of 0.87 with a specificity of 0.92 for an 8 ppm detection threshold [76]. Improvements to performance could be achieved through requiring a response on more than one indicator material. Unfortunately, this algorithm failed when applied to data collected using the housed prototype device (Figure 3b). In the initial experiments, petri dishes were used to expose the indicator materials to targets [74]. These types of exposures have little relevance to the expected target concentration changes for an environmental sensor system. Exposure of the device using the glove chamber was intended to better simulate the application conditions [74]. These exposures produced a significantly different time dependence in the change in color values for the indictors. The differences reflected the time required to reach peak concentration and the slow return to baseline conditions. The standard deviation algorithm for the dataset collected using this approach yielded sensitivity 0.07 with specificity 0.99. Manual analysis of the data, on the other hand, indicated that detection of ethanol at 160 ppb should be possible.

### 4.2. Slope Algorithm

**Figure 5:**
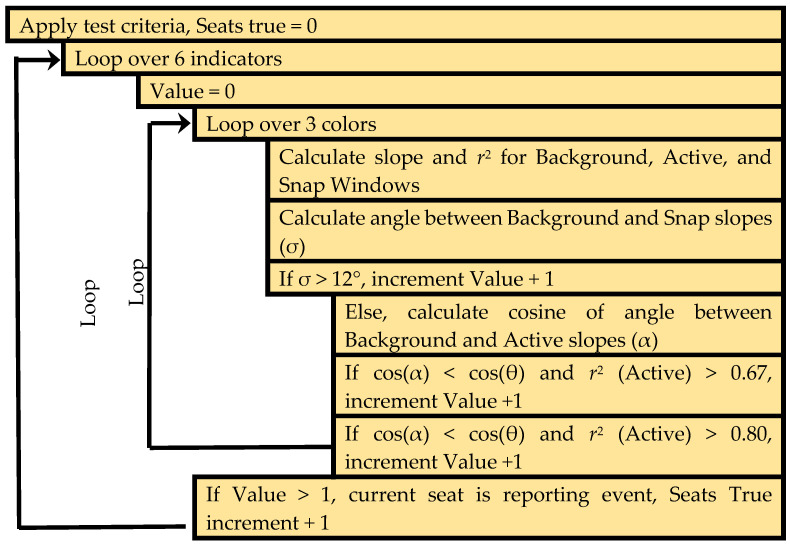
Pseudocode describing the sloped-based algorithm.

Given the failure of the standard deviation based algorithm when used with environmentally relevant exposures, an additional algorithm was developed [76]. Slope-based detection criteria were used again based on examination of the red, green, and blue color channels for each of the six indicators (Figure 5). Here, the thresholds for detection were fixed for each color value of each indicator based on data collected during device warm up, the first 120 data points following initiation of the device. The initial intensity was used in the following formula:

(1) $$\emptyset =ae^{\frac{-RGB}{b}}+c$$

where *RGB* was the initial intensity value for a given color channel. The parameters used in this approach depended on the minimum number of indicators required for a detection event or on the initial intensity values: *a* = 20 for one indicator or 70 for more than one and *b* = 130 for one indicator or 30 for more than one. The value of c was equal to the larger value of two possible values: A user specified value (default 0.45°) or the algorithm calculated angle of the dot product of the standard error for the 120 data points collected during initiation of the device. These parameters were optimized using a set of controlled exposures in the enclosure experiments.

For event detection, linear regression was used to compute the slope and *r*^2^ value for each of the colors (RGB) over two time windows, Active and Background. The Active window was the 20 most recent time points; the Background window was populated by the next 120 most recent time points. These windows were used for calculation of the cosine of the angle between the slopes. Each of the three color values was considered for each indicator, and the value was compared to the cosine of the Threshold angle determined above. If the cosine of the angle between the Background and Active slopes was less than that of the Threshold angle and the *r*^2^ value for the Active window was greater than 0.67, the color value was counted as 1. If the *r*^2^ value was greater than 0.8, that color was counted as 2. The counts were summed for a given indicator; a value greater than 1 led to that indicator being considered to have detected an exposure [98]. Rapid changes resulting from high dose exposures were captured by an additional test. The Snap window contained only the 10 most recent data points. If the angle between the Snap slope and the Background slope was greater than 12° then the RGB channel contributed 1 to the running total. As with the standard deviation algorithm, an event window was used for determination of the end point; 60 min in this case.

## 5. Demonstration and Evaluation

**Figure 6:**
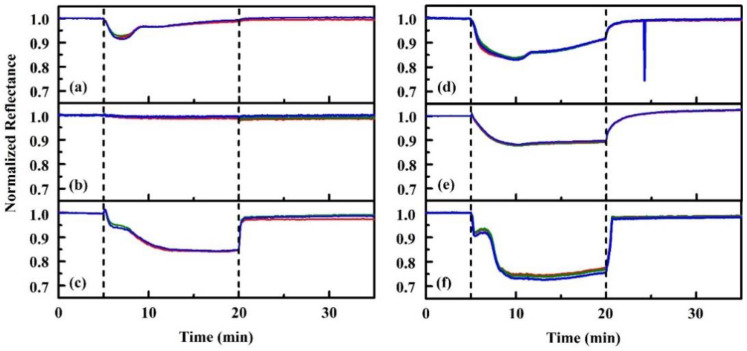
Porphyrin responses to stagnant alcohol exposures (0.5 mL ethanol). Shown here are time-dependent responses for the porphyrin functionalized WypAll materials upon exposure to targets under the petri dish method: (**a**) Deuteroporphyrin IX bis ethylene glycol (DIX), (**b**) meso tetra(4-aminophenyl)porphyrin (N_4_TPP), (**c**) Cd DIX, (**d**) Ce DIX, (**e**) Co DIX, (**f**) Fe DIX. Dashed lines indicate the beginning and end of the exposure duration. Data have been normalized to the average total signal during the initialization period.

Initial work was completed using the earliest, unhoused device (Figure 3a) [74]. These tests used custom Delrin holders, designed to sit as the lid on a 60 mm Petri dish, to screen indicators and evaluate the performance of the color sensing devices. Exposures were completed by first collecting baseline data over an empty Petri dish (60 mm; total volume 57 mL). The holder was then moved to a warmed dish containing 1 mL or less of an alcohol target. Following exposure, the holder was moved back onto the empty dish to monitor changes as the indicator returned to baseline. Figure 6 provides an example of the data resulting from one such experiment. These evaluations were used to identify indicator materials that showed promise for use in an alcohol (isopropanol, ethanol, methanol) detection array. Alcohols were selected for much of this work because the low hazards associated with their use provided flexibility in the types of exposures and environmental conditions that could be considered. In total, 130 porphyrin and metalloporphyrin indicators were screened for the three alcohol targets at five exposure levels. Based on the results, a set of six materials was selected: N4TPP, silver (Ag N4TPP) and zinc (Zn N4TPP) variants of N4TPP, and silver (Ag DIX), yttrium (Y DIX), and thallium (Tl DIX) variants of Deuteroporphyrin IX bis ethylene glycol. The indicators were selected based on their providing varied response across the three targets and on the scale of the intensity changes upon exposure. Both exposure indication and target discrimination were desired in this array.

**Figure 7:**
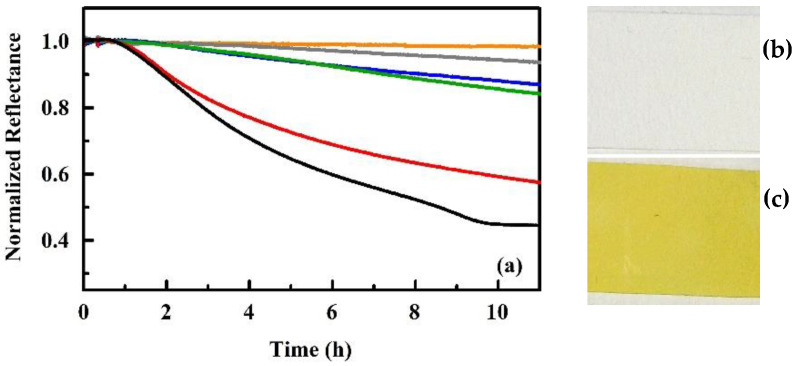
Paper supported titanyl indicators. (**a**) Shown here are the normalized blue color values for indicator swatches before and during exposure to vapor evolving from water (orange) and 3% (gray), 1.2% (blue), 0.3% (green), 0.15% (red), and 0.06% (black) solutions of hydrogen peroxide in water. Pictured here are the fresh (**b**) and exposed (**c**) titanyl indicators [97].

A similar type of experimental setup was used to evaluate both porphyrin and titanyl indicators intended for the detection of peroxides related to illicit explosives manufacture [97]. While the prototype device reports RGB color values, the changes observed for the titanyl indicators were negligible on the red and green channels. The rates of change in reflectance for the titanyl indicators were found to be concentration dependent (Figure 7). The initial work with porphyrin indicators focused on reversible, chemosorptive interactions. The titanyl indicators were reactive and non-reversible (Figure 7), demonstrating the potential for use of the prototype device with other types of indicator materials. The porphyrin indicators (Co DIX and Cu DIX) provided reversible changes in reflectance upon exposure to peroxides. They also provided responses upon exposure to sulfuric, nitric, and hydrochloric acid [97].

**Figure 8:**
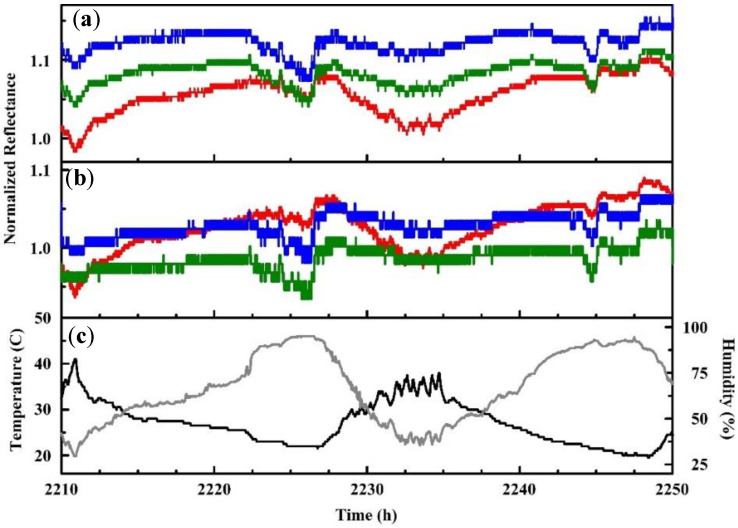
Red, green, and blue (RGB) profiles for data collected in an outdoor environment for a period between 6 and 8 July 2015: (**a**) Zn N4TPP and (**b**) Ag DIX. (**c**) The temperature (black) and humidity (gray) recorded by a co-located data logger are also provided [75].

The Delrin housed devices (Figure 3b) were used for a large number of evaluations including those completed in outdoor environments and in a glove chamber (65 L; Techni-Dome, Bel-Art, Wayne, NJ, USA) [75,76]. The enclosure was used to complete screening evaluations similar to those completed with the unhoused device as well as to evaluate additional targets. The six-element array identified above (N4TPP, Ag N4TPP, Zn N4TPP, Ag DIX, Y DIX, and Tl DIX) was used for collection of several large datasets. The majority of the work utilized three prototypes running simultaneously. In indoor experiments, they were contained in a single environment for collection of 3771 h of continuous operation (data were downloaded once per week). Outdoor experiments used the three prototypes in three different locations for collection of 19,597 h of data (Figure 8). Both types of experiments included exposures to alcohols at various points during data collection. Initial assessments of the datasets indicated a difference indicated a significant loss in specificity (based on receiver operating characteristics, ROC) when the devices were moved from indoor to outdoor environments. Adjustments to the algorithm were made in an attempt to address these differences. Specificity could be improved using changes to the detection thresholds or the minimum indicator requirements but only at the expense of sensitivity [75].

**Figure 9:**
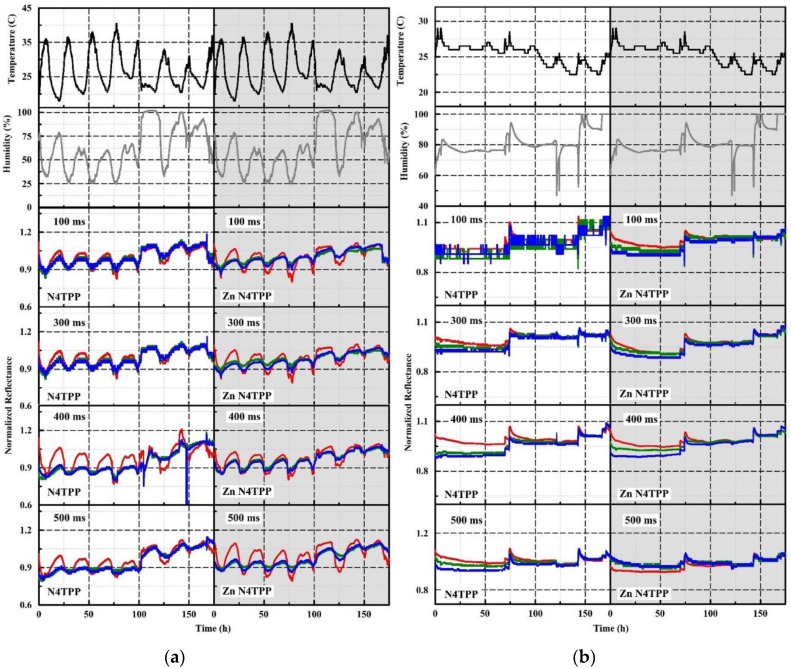
Normalized RGB color value dataset collected for N4TPP and Zn N4TPP in the laboratory (**a**) and in an outdoor environment (**b**) comparing 100, 300, 400, 500 ms integration for the prototype devices. Temperature (black) and humidity (grey) are reported using a co-located device [75].

A follow-up experiment considered the potential impact of adjusting device parameters. Additional data (1148 h indoor, and 1056 outdoor) was collected for comparison of different device integration times (Figure 9) [75]. The initial datasets were collected at 100 ms with indoor sensitivity of 0.5 and specificity 1.0. The 400 ms indoor dataset yielded ROC specificity and sensitivity of 1.0 under a single indicator requirement. Similar outdoor data, however, yielded high false positive rates under the single indicator requirement. A variation of the algorithm requiring three indicator responses and using a reduced detection threshold yielded specificity 0.94 and sensitivity 0.83 (300 ms data). Further improvements to false positive rates could be made only through sacrificing sensitivity. Including untreated WypAll as one of the indicator materials (a negative control) further improved performance with specificity 0.97 and sensitivity 1.0. In the optimized approach 145 h of outdoor data provided a run time of 70 h before a false response was detected.

Independent evaluation of the housed prototype was completed, including exposure to a series of previously unevaluated targets (Table 1) [99]. These evaluations used six indicators materials: N4TPP, Ag N4TPP, Zn N4TPP, Au DIX, Y DIX, and Tl DIX, basing the selections largely on the initial array described above. The evaluations required some alterations to the way the algorithm was implemented to meet the requirements of the evaluation methodology. Previously, 120 data points were used to populate the initial Background window with an additional 20 points required to populate the Active window. A shorter warm-up period was required for these evaluations. To accommodate this need, the initial 30 data points were entered into the buffer in four positions. The data used by the algorithm have the same data at 0, 30, 60, and 90 s, the data collected at time = 0 s. The data at 120, 150, 180, and 210 s is replication of the data collected at the 30 s time point. At 15 min, entry of data into the algorithm returns to normal. One other alteration was made, where the cool down period was changed from 120 points (60 min) to 10 points (5 min).

The changes made to the algorithm in combination with the way the devices were evaluated had a negative impact on the outcome of the experiments [99]. A recurring event was noted at approximately 1500 s (0.42 h). Based on the changes made to the way the algorithm windows were populated, this was the first time point at which detection could begin following the 25 min warmup period. In this set of experiments, the fans of the prototype were used to circulate the air within the test setup; air flow was initiated at the time that data collection began. The associated humidity data shows a steep change through the first hour of data while the test chamber is equilibrated. The initial Background window for the algorithm was populated by data reflecting the rapid humidity change, and the changing slope caused an event trigger when the detection algorithm came online. It is also important to note that the threshold angles for each color of each indicator and the associated standard deviations were fixed by the first 120 points in the matrix; this was the only calculation of those values for a given use cycle. This calculation defined the sensitivity of the algorithm; large changes during this period had a negative impact on performance.

**Figure 10:**
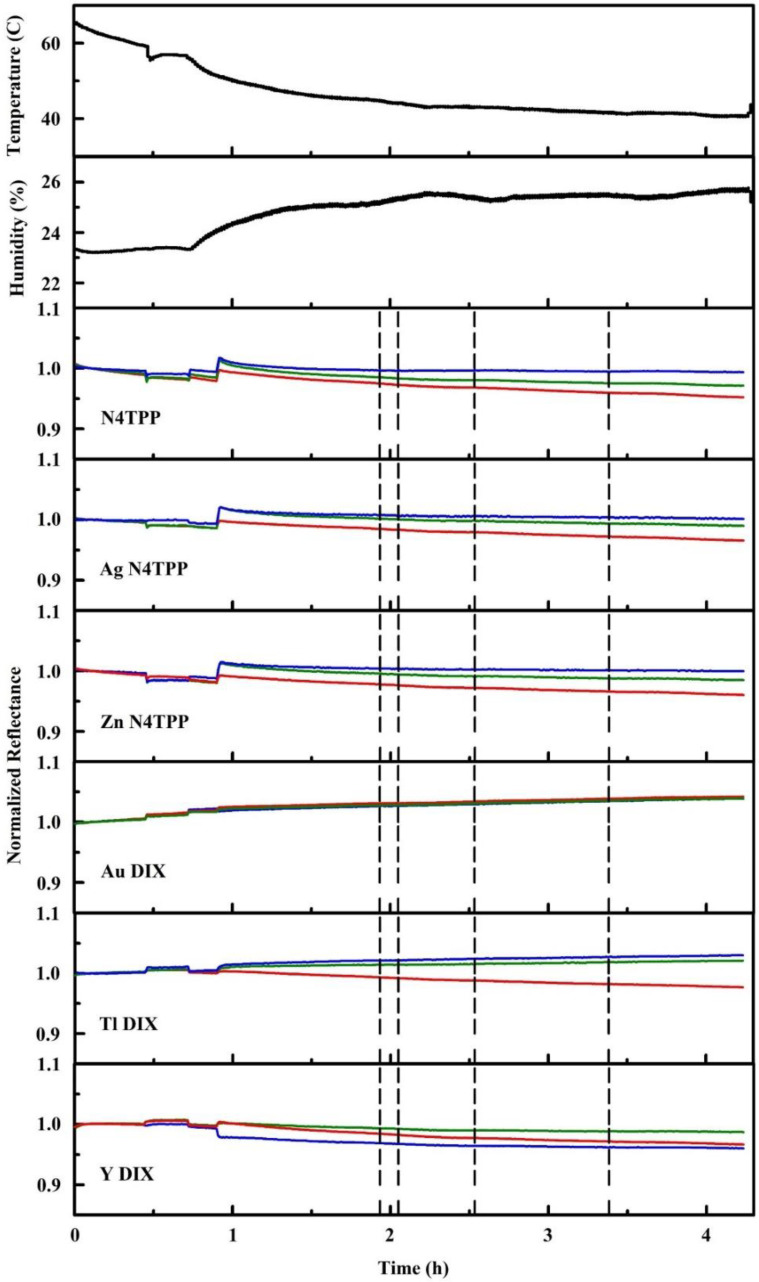
Ethylene oxide exposures at 78 ppm. Dashed lines indicate the beginning of chemical stream flow. Humidity and temperature data collected during the experiment are also provided [99].

Table 1 provides two sets of algorithm responses. The first is based on the reported events provided by the dripfeed algorithm during the experiments. The second set used a modified algorithm after experiments were completed [99]. Here, we take exposure to ethylene oxide as an example of the data collected during this set of evaluations (Figure 10). The prototype device was exposed to 78 and 361 ppm ethylene oxide vapor. Events were noted prior to the beginning of exposures as described above. For a total of 15 exposures, a single exposure associated detection event was noted.

**Figure 11:**
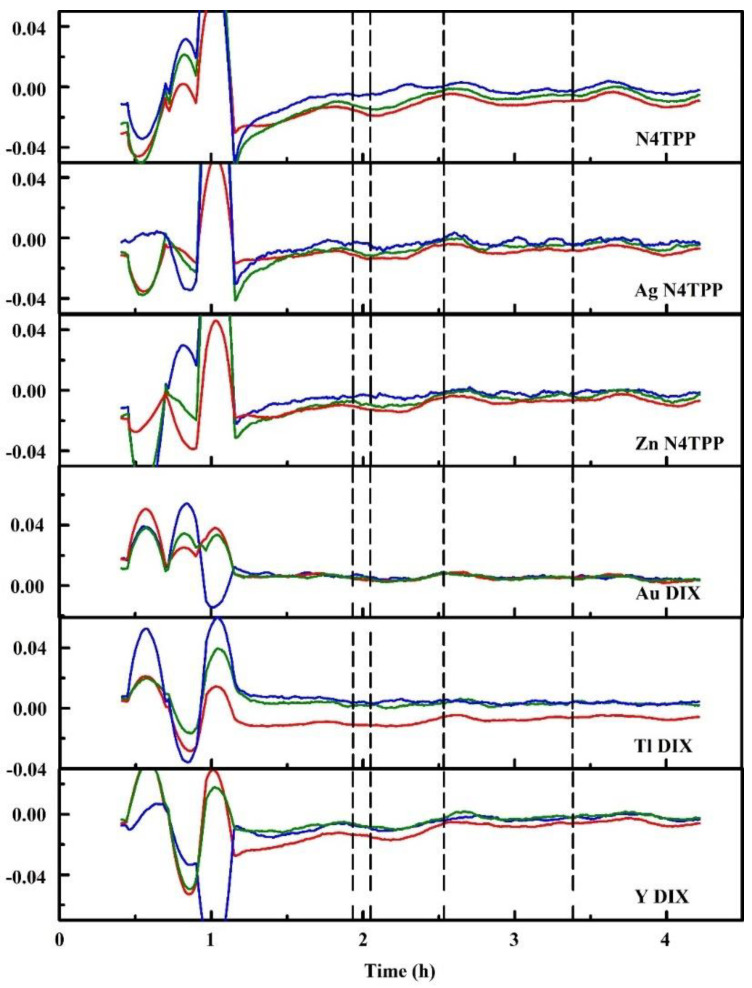
Slopes for ethylene oxide exposures. Here, a 30-point sliding window is applied to calculation of the slope. Prior to slope calculation, intensity values are normalized to the first intensity value for each color of each indicator. Dashed lines indicate the beginning of chemical stream flow, 78 ppm ethylene oxide [99].

Alteration of a new detection algorithm without sufficient datasets risks overtraining where the device and algorithm have been tuned to respond well under test conditions that may not accurately reflect other applications. This was observed for the initial algorithm development described above [99]. The data generated during this test of the prototype were not sufficient for generation of a new automated algorithm. The intrinsic response of the device was, however, examined using the slopes of the collected data over time. This was a manual investigation, but it provided a better idea of the possible response profiles that the algorithm was not designed to capture (Figure 11). The complication of early data points became apparent in this dataset. The low concentration exposures produced little response from the device; higher concentration exposures produced characteristic responses from the N4TPP, Ag N4TPP, Zn N4TPP, and Tl DIX indicators. Adjustment of the detection thresholds (again, a manual analysis), indicated that it should be possible to improve detection of ethylene oxide and the other targets (Table 1) [99].

During the course of these experiments, the device suffered several failures [99]. The power plug connection was damaged through user interaction, requiring replacement of the control board. During board replacement, the connector on the control board for one of the color sensors was damaged. A final failure of the control board was found to be a result of corrosion on the main board. This corrosion followed exposure to Cl_2_, a corrosive gas. The result of these failures was an incomplete dataset; however, a significant amount of data were available. As shown in Table 1, the prototype was able to detect a number of the compounds used, but the results were mixed. This detection occurred with a non-optimal algorithm implementation, during multiple device failures, and without an optimized set of indicator materials. Significantly improved performance would be expected if these issues were addressed.

## 6. Ongoing Work

While the prototype system described in this review was proven to provide detection of targets under the desired conditions, it fell short in many aspects. Six indicators were insufficient for real-world applications. A larger array would allow for better target discrimination and avoidance of false positive/negative responses. The openings in the current housing, necessary for power and communications, make isolation of the electronics difficult, leading to failures resulting from long-duration environmental exposure as well as upon exposure to corrosive targets [99]. Finally, the device did not include onboard power or remote communication capabilities (Table 2). These lacking capabilities prevent the use of the devices in a distributed network, requiring USB tethering to a laptop computer or other supplementary data logger for communications.

**Figure 12:**
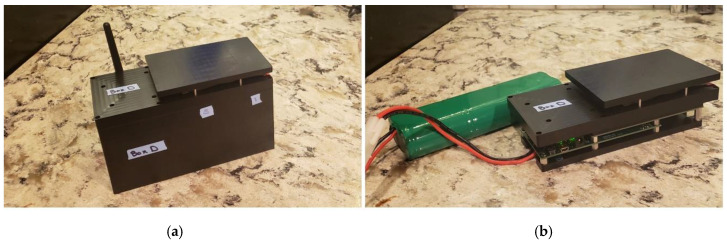
(**a**,**b**) Images of the ABEAM-15 prototype sensor. The device includes fifteen surface mount RGB sensors, eight cool white LEDs, and three printed circuit boards: The bottom control board, the middle board with a 5 × 3 array of color sensors, and the top illumination board. This device can be utilized wirelessly for real-time detection.

Based on lessons learned with the initial prototypes and keeping in mind the desire to design a distributed network, a new prototype device is being evaluated (Figure 12) [100,101]. This device is intended to provide the necessary expanded instrument capabilities while retaining similar size, weight, and power (SWaP) characteristics at a manageable cost. The physical geometry of the instrument is crucial to this design. Here, the intention was to increase the number of indicators without significantly changing the size of the device. This requires a completely different approach to the optical design as well as a different approach to design of indicator coupons; a single coupon bearing all of the indicators is necessary to accommodate the smaller footprint. The data sampling rate and the autonomous deployment duration are also important. With an increase in the number of indicators, each sampling cycle generates significantly more data than that of the six-element array. Physical isolation of the instrument electronics from the environment, incorporation of wireless communications, and onboard power/power management were also priorities.

In this new device, the older TCS3200-DB breakout boards have been replaced with TCS34725 surface mount RGB sensors. Rather than providing a pulse train proportional to intensity, these output a voltage proportional to intensity of the signal. This allows for higher data throughput, resulting in faster sampling cycles even with the increased number of array elements. As an example, the original TCS 3200-DB breakout board was limited to 100 ms integration for a 5 s sampling cycle. The current design (ABEAM-15) can perform the 5 s sampling cycle at integration times of up to 600 ms. Software for this device has been re-written in Java. The user interface was re-written using JavaFX, removing many of the limitations of the old LabWindows based GUI. Experimental modes have been expanded to include autonomous operation with post collection analysis, drip-feed analysis, and a distributed microsensor network mode utilizing a star-point topology with real-time analysis. The detection algorithm used to identify the occurrence of events has not changed, but the implementation has been updated. The devices can be used singly or as a network containing up to six devices controlled by a laptop computer.

## 7. Conclusions and Future Outlook

Here, we have provided a review of the hardware and algorithm development for a prototype sensor system intended for distributed autonomous applications. Through several device generations, evaluations have included a wide range of conditions and scenarios: Laboratory based screening and longer duration indoor evaluations, independent laboratory evaluations, and outdoor deployments. While the devices offer demonstrated capabilities directed toward the desired distributed application, the ABEAM-6 instrument falls short in incorporation of necessary power and communications capabilities as well as in the total number of indicator materials. The 15-element array currently under evaluation offers significant improvements in capability over that of the original six-element device, but there are advantages to be found in further expansion of the number of array elements. More elements offer the potential for improved discrimination of targets as well as data redundancy, a feature that can improve confidence. It may also be possible to incorporate different types of indicator materials in a larger array, providing greater insight into target concentrations.

In redesigning the prototype to both incorporate 24 elements and provide long-duration unattended operation, the spacing of the detection elements must be considered. In the 15-element device [100], indicator spots are located 14 mm apart in a five-by-three array. It is likely possible to reduce both the size of the spots and the spacing between them, but the limitations will have to be determined. A change in the illumination of the spots will also be considered; in the current layout, some of the indicators are illuminated by multiple overlapping LED patterns while others are illuminated by a single LED. It would be preferable to have uniform illumination of all indicator materials. Evaluations of the current system have demonstrated inefficiencies leading to faster than predicted battery drain and highlighting the need for adjustments to available battery power and power management. Addition of increased battery capacity would also increase size and weight, but improvements should be possible through redesign of onboard power management strategies. Beyond these considerations, the flash memory is undersized for long durations of onboard storage. Incorporation of these types of modifications should facilitate expansion to 24 indicator elements while supporting further reductions in size and maintaining the sampling frequency of the 15-element device.

The general sensing approach described here has been used previously; however, applications were in dosimetry or in end-point type detection [44,61]. Much of the related work also relies on exposure of the indicators followed by removal and analysis by image capture, an approach requiring significant processing power and time. The prototype devices described here are directed at long-term, autonomous monitoring of changes in the chemical composition of an environmental air sample, an application requiring significantly different performance characteristics. The devices for this application must provide long-duration unattended function. While additional development is necessary to meet the goals of the application, this review provides a summary of progress to date and demonstrates the feasibility of the sensing concept and its potential strengths and weaknesses through a wide range of evaluations and demonstrations.

## 8. Patents

Patent application: US 2017/0343471 30 November 2017 “Method for Analysis of Data Related to Use of Reflectance Based Color Changes in Real Time Sensing Applications,” A.P. Malanoski, B.J. White, J.S. Erickson, D.A. Stenger.

US 9,581,594 28 February 2017 “Porphyrin-modified antimicrobial peptides for application as indicators of microbial targets,” C.R. Taitt, B.J. White.

## Figures and Tables

**Table 1:** Performance summary [99].

Target	Total Exposures	Initial Algorithm Events	Altered Algorithm Events ^1^
Ethylene oxide, 78 ppm	9	1	5
Ethylene oxide, 361 ppm	6	0	5
Simple Green	6	3	0
Sarin, 0.22 mg/m^3^ (Simple Green)	7	1	6
Sulfur Mustard, 1.2 mg/m^3^	6	0	3
Sulfur Mustard, 2.5 mg/m^3^	7	5	7
Chlorine gas, 5 ppm	6	6	6
Chlorine gas, 100 ppm	7	4	4
VX, 0.013 mg/m^3^	6	0	4
VX, 0.022 mg/m^3^	6	0	5

^1^ Based on post-processing of data collected.

**Table 2:** Device summary.

Device	Multiplex	ABEAM-6	ABEAM-15
Number of Indicators	6	6	15
Memory Duration (30 s sampling)	7.5 days	60 days	12 days
Size (LxWxH)	Not housed	27.4 × 7.6 × 7.6 cm	19.1 × 8.9 × 11.7 cm
Weight	450 g	1.6 kg	2.2 kg ^1^
Software Platform	LabWindows	LabWindows	Java/JavaFX
Sensor Hardware	TCS 3200	TCS 3200, 3400, 34725, and AS 7262	TCS 34725
5 s Integration Times	100 ms	100–500 ms ^2^	100–600 ms
Available Gain	No	Some ^3^	Up to 64×
Microcontroller	ATMEGA 328-P	XMEGA64-A3U-AU	XMEGA64-A3U-AU
Wireless Communications	No	No	Yes
USB Communications	Yes	Yes	Yes
Power Management	No	No	Yes
Fans	No	Yes	No
Outdoor Housing	No	Yes	Yes
Batteries	No	No	Yes
Drip feed, Real-time Detection	No	Yes	Yes
Networkable	No	No	Yes
Dimmable LEDs	No	No	Yes

^1^ Includes batteries. ^2^ The ABEAM-6 offers up to 500 ms of integration time on a 5 s collection cycle for the TCS 3414 sensor hardware only. All other ABEAM-6 sensor hardware offers 100 ms only. ^3^ All sensors except the TCS 3200 offer gain of up to 64×.

## Supplementary Materials

The following are available online at https://www.mdpi.com/1424-8220/20/20/5857/s1, Figure S1: Block diagram for the ABEAM-6 prototype sensor [101], Figure S2. The ABEAM-6 control board is 7.0” long × 2.0” wide (17.78 cm × 5.08 cm). Selected components and dimensions have been labeled. (a) Front side. (b) Back side. (c) Control board mounted in a partially assembled housing (top view). Two fans and six Parallax TCS3200-DB sensors have been added. The cables that connect the sensors to the board have been removed for clarity [101].
