# Supplementary material for: Development of a Colorimetric Sensor for Autonomous, Networked, Real-Time Application
Source: Sensors (Basel). 2020 Oct 16;20(20):5857. doi: 10.3390/s20205857 (PMC7589661; doi:10.3390/s20205857)
Supplement: Supplementary file 1 [file sensors-20-05857-s001.pdf]

sensors

Supplementary Information

Development of a Colorimetric Sensor for Autonomous, Networked, Real-Time Application

Brandy J Johnson *, Anthony P Malanoski and Jeffrey S Erickson

Center for Bio/Molecular Science & Engineering, US Naval Research Laboratory, Washington, DC 20375, USA; anthony.malanoski@nrl.navy.mil (A.P.M.); jeffrey.erickson@nrl.navy.mil (J.S.E.)
* Correspondence: brandy.white@nrl.navy.mil; Tel.: +1-202-404-6100

Received: 15 September 2020; Accepted: 9 October 2020; Published: date

This supporting information provides additional detail on the ABEAM-6 prototype sensing devices including additional images and a block electronics diagram.

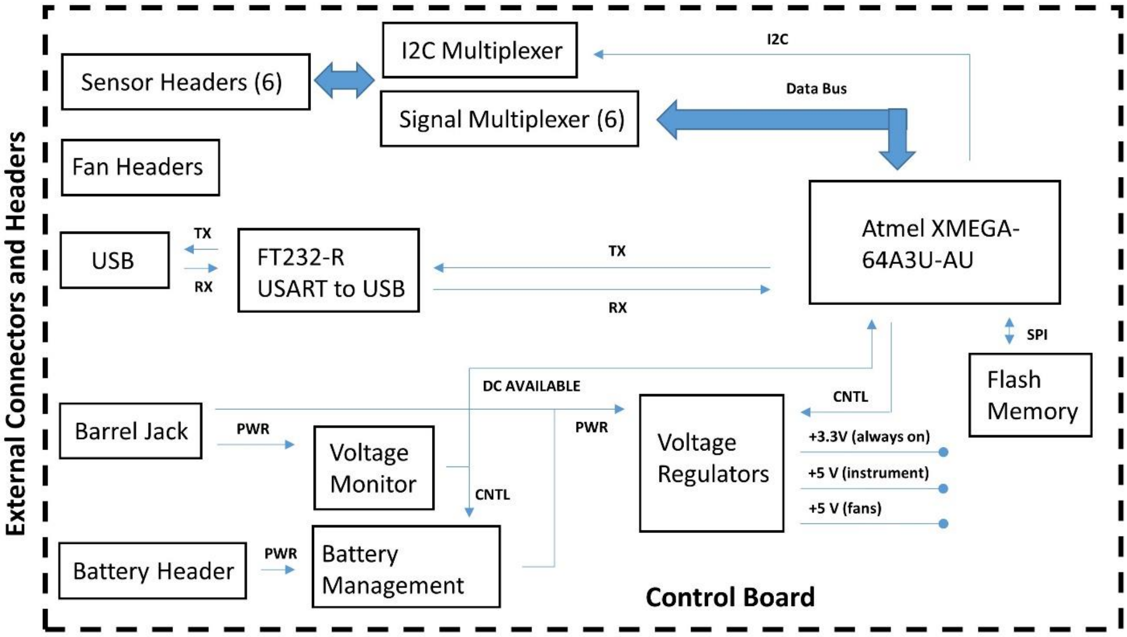

Figure S1. Block diagram for the ABEAM-6 prototype sensor [101].

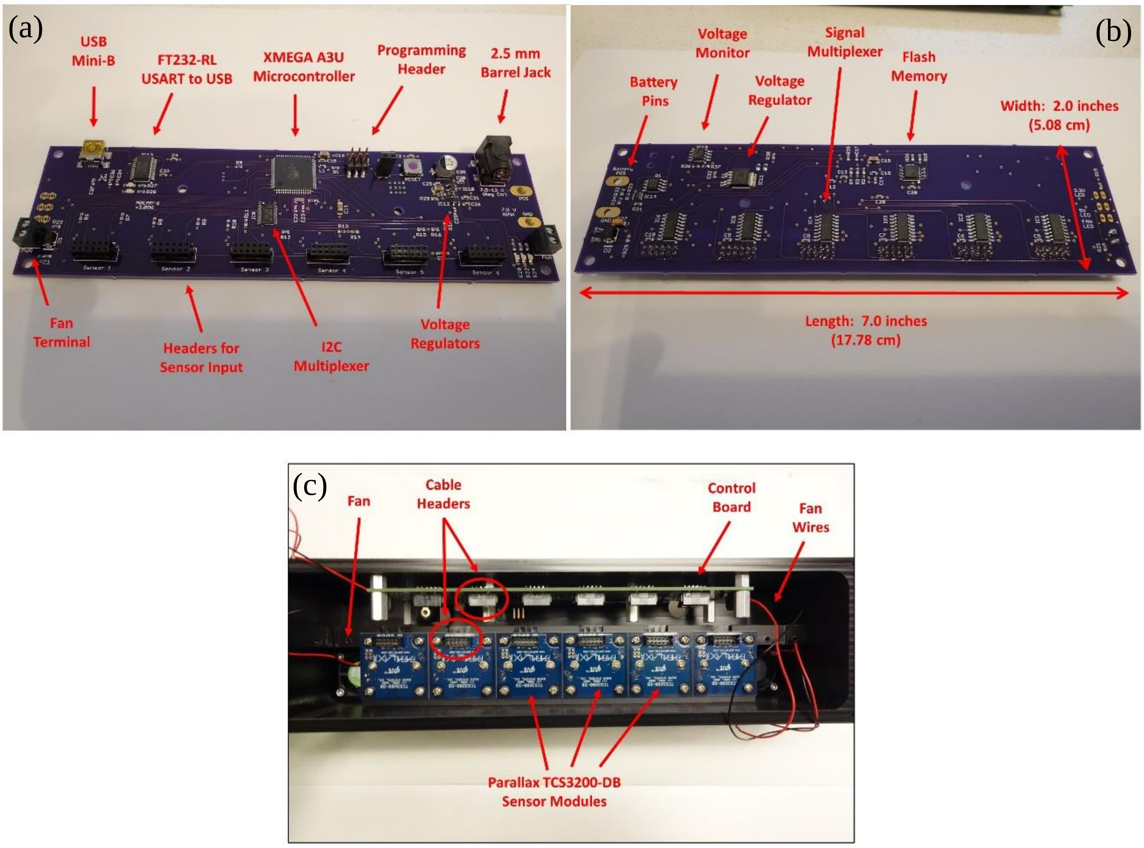

Figure S2. The ABEAM-6 control board is 7.0″ long × 2.0″ wide (17.78 cm × 5.08 cm). Selected components and dimensions have been labeled. (a) Front side. (b) Back side. (c) Control board mounted in a partially assembled housing (top view). Two fans and six Parallax TCS3200-DB sensors have been added. The cables that connect the sensors to the board have been removed for clarity [101].

© 2020 by the authors. Submitted for possible open access publication under the terms and conditions of the Creative Commons Attribution (CC BY) license (http://creativecommons.org/licenses/by/4.0/).
